# Prevalence and factors associated with the use of modern contraceptive methods among female healthcare providers in health facilities in Guinea

**DOI:** 10.3389/fgwh.2025.1567960

**Published:** 2025-06-19

**Authors:** Yamoussa Youla, Sidikiba Sidibé, Hounmenou GC, Mory Kourouma, Saidouba Chérif Camara, Salifou Talassone Bangoura, K. K. J. J. Olivier, Mathias Grovogui, Maladho Diaby, Emile Faya Bongono, Oumou Hawa Diallo, Ibrahima Conté, Sékou Traoré, Adama Djigui Keita, A. Touré, A. Delamou

**Affiliations:** ^1^ Department of Pharmaceutical and Biological Sciences, Faculty of Health Sciences and Technology, Gamal Abdel Nasser University, Conakry, Guinea; ^2^ Centre de Recherche et de Formation en Infectiologie de Guinée, Université Gamal Abdel Nasser de Conakry, Conakry, Guinea; ^3^ Department of Medical Sciences, Faculty of Health Sciences and Techniques, Gamal Abdel Nasser University, Conakry, Guinea; ^4^ IT Department, Centre Universitaire de Labé, Labé, Guinea; ^5^ Maférinyah Rural Health Research Centre, Ministry of Health and Public Hygiene, Forecariah, Guinea

**Keywords:** modern contraceptive method, use, Guinea, prevalence, associated factors

## Abstract

**Introduction:**

The low use of modern contraceptive methods by female healthcare providers is a real public health problem in Guinea. This study aimed to analyze the prevalence and factors associated with the use of modern contraceptive methods among female healthcare providers in Guinea.

**Method:**

This was a secondary analysis of data from a cross-sectional survey on the use of modern contraceptive methods among female healthcare providers in Guinea between November 1, 2020, and January 31, 2021. The study included 1,743 women from 173 health facilities across the country.

**Results:**

This survey included women whose median age was 29 years, with an interquartile range of (24–39) years. Women with a higher level of education (*n* = 1,656; 95.5%) and a midwifery profile (*n* = 838; 48.3%) represented the highest proportions. Among the subjects surveyed, Muslim women were the majority (*n* = 1,229; 70.9%). The overall prevalence of use of modern contraceptive methods by female healthcare providers in Guinea was 61.9%; CI: 59.6–64.1). Married (aOR = 1.19; CI = 0.95–1.50), having secondary education (aOR = 7.92; CI = 3.68–20.7), women belonging to the Muslim religion (aOR = 1.37; CI = 1.09–1.71) were factors statistically associated with the use of modern contraceptive methods among female healthcare providers.

**Conclusion:**

Improving the national prevalence of modern planning methods requires the involvement of stakeholders at all levels. This study's results show a progression in the implementation of governmental actions and health projects and programs related to sexual reproductive health.

## Introduction

From 2015 to 2019, every year, 121 million unwanted pregnancies were counted, of which 61% ended in abortion (1). In 2021, 1.1 billion of the world's 1.9 billion women aged between 15 and 49 were in need of family planning services. Of these, 874 million were using modern contraception, while 164 million were denied access to contraception when they needed it (2). The proportion of women of childbearing age using modern family planning methods is one of the indicators in point 3.7.1 of the Sustainable Development Goals. In recent years, significant progress has been made worldwide in improving contraceptive prevalence among women of childbearing age 15–49 (3). According to the World Health Organization (WHO), contraceptive prevalence rose from 74% to 77% worldwide among women of childbearing age between 2000 and 2020 (4). The proportion of women of childbearing age (15–49) using modern family planning methods was 77.5% worldwide in 2022 (5–7). Despite this progress, disparities persist in access to and use of modern contraceptive methods between different regions of the world (8). In high-income countries, over 70% of women have access to a contraceptive method (9–11). In Africa, where the burden of maternal mortality is highest, barely 24% of women of childbearing age have access to a modern contraceptive method (4). In these countries, more than half (56.0%) of modern contraceptive needs remain unmet (12).

Pregnancies that are too early, too late, too numerous and too close together are responsible for the majority of direct obstetric complications, which account for over 70% of maternal deaths in low-income countries (13, 14). Family planning (FP) can prevent 35% of maternal deaths in poor countries if at least 60% of women of childbearing age use a contraceptive method (7).

In their study of the Democratic Republic of Congo, ZIVICH et al. reported a prevalence of 48% of women using contraception (7, 10, 15). In Burkina Faso in 2023, the prevalence of use of modern contraceptive methods was 18.1% (16). In Mali in 2020, out of a total of 2,097 registered users, 721 women (31.38%) were new users of modern contraceptive methods (17).

Few studies have been conducted on the use of contraceptive methods among female healthcare providers in Africa. In northern Uganda, contraceptive prevalence among women working in the health sector in teaching hospitals was 73.6% (16). This prevalence was 18% at the time of the survey of healthcare professionals and medical students in their clinical year in Ghana (17).

Several factors influence the use of contraceptive methods in the general population in sub-Saharan Africa. The main positive factors are education level, family planning knowledge, multiparity and high wealth index (8, 16–18). The negative factors that reduce contraceptive use are fear of contraceptive side effects, male partner disapproval and socio-cultural norms (16). In a study conducted in Uganda, patient- and provider-related barriers to contraceptive use were identified. Patient-related barriers included religious beliefs, misconceptions about contraception and apprehension about adverse effects. Difficulties associated with providers included lack of knowledge, inadequate training and discomfort (19).

In Guinea, contraceptive prevalence is still low among women of childbearing age (15–49). According to the Demographic and Health Surveys (DHS) results, it rose from 9.0% in 2012 to 11.0% in 2018 (20). Furthermore, most of the studies carried out on contraception in Guinea have focused on adolescent girls and young women. There has been a modest increase in modern contraceptive use among adolescents and young women, from 8.4% in 1999 to 12.8% in 2018 (21). Only 22.6% had unmet contraceptive needs (22). The main factors influencing the use of contraceptive methods were fear of adverse effects, spousal approval, religious and socio-cultural beliefs, and the skills of healthcare providers (21, 23) and women from affluent households (21). No nationwide study has explored and documented the use of modern contraceptive methods among female healthcare providers, hence the interest in carrying out this work. The aim was to analyze the prevalence and factors associated with the use of modern contraceptive methods among female healthcare providers in Guinea.

## Method

### Study setting

**Figure 1:**
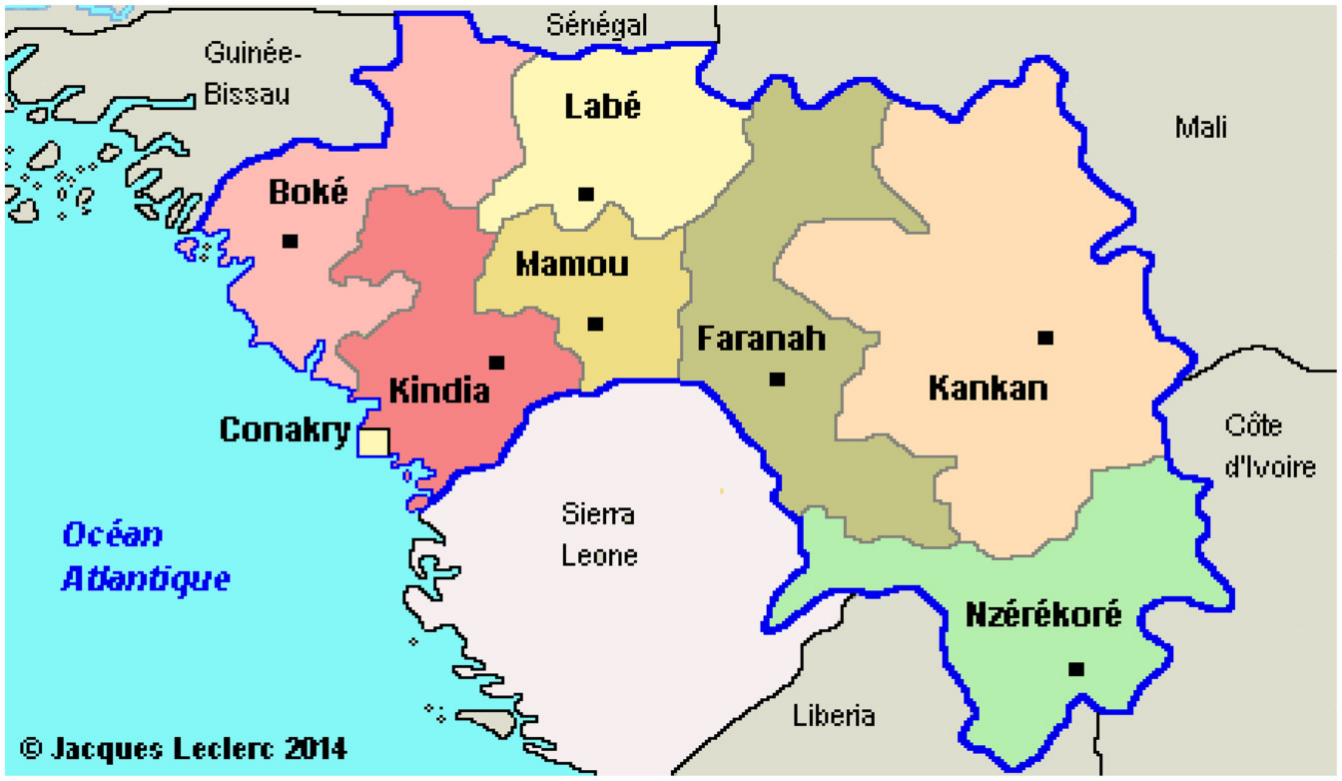
Geographical map of Guinea's eight (8) administrative regions and neighbouring countries.

The study was conducted in public and private health facilities [hospitals, communal medical centres (CMC), health centres, and clinics] in the eight (8) administrative regions of Guinea (Figure 1) between November 1, 2020, and January 31, 2021.

### Type, period and study population

This was a secondary analysis of data from a cross-sectional survey on the use of modern contraceptive methods among female healthcare providers in Guinea between November 1, 2020 and January 31, 2021.

The study covered all female healthcare providers (doctors, nurses, midwives, technical health agents, laboratory technicians, pharmacists) working in Guinea's public or private healthcare facilities that provide FP services.

### Data sources

Data for this study were extracted from the database of a cross-sectional survey conducted in 2021 across 173 public and private health facilities. These included 19 communal hospitals/medical centers (CMCs), 69 health centers, 41 private hospitals/clinics, and 54 private practices or health centers located in the capitals of the eight administrative regions of Guinea, including the capital city, Conakry. A multi-stage sampling technique was used to select the health facilities and study participants. First, an exhaustive selection of national and regional public hospitals and communal medical centers (CMCs) was conducted in each region. Second, a simple random sampling procedure was applied to select among the remaining eligible health facilities (i.e., health centers, private clinics, medical practices, and NGOs), stratified by public or private status, using a validated list provided by the Ministry of Health. Among the 328 eligible health facilities identified, 173 (52.8%) were selected to participate in the study. Within each selected facility, all female health care providers present on the day of data collection were invited to participate. Female providers with missing data on outcome variables were excluded from the analysis. This sampling strategy was designed to ensure representativeness across regions, types of facilities, and public-private distribution.

### Dependent or outcome variable

The primary study variable was the current use of a modern contraceptive method, which was coded as a binary variable: “yes” for providers (respondents) who reported using a modern method of contraception at the time of the survey, and “no” for those who were not using any modern method, including those who were using traditional methods. Modern FP methods were defined as intrauterine devices (IUDs), implants, injectables, contraceptive pills, sterilization (male and female) and male and female condoms.

### Independent variable

Independent variables included sociodemographic characteristics such as age at the survey, region of residence, marital status, religion, level of education and occupation. They also included work experience, type of contraceptive method used, work in a family planning service, and participation in family planning training.

The level of education included no education, primary, secondary, and higher education. Religion had two categories: Muslim and Christian/other. Marital status was either single or already married; regions were classified as Conakry, Kindia, Boké, Mamou, Labé, Kankan, Faranah, and N'Nzérékoré.

### Statistical analysis

Data were analyzed using R-Studio software version 4.4.1. Descriptive statistics were produced as proportions for qualitative variables and mean with standard deviation (SD) for quantitative variables. The Chi-2 statistical test was used to compare qualitative variables, and the Wilcoxon test for quantitative variables in the univariate analysis. Logistic regression was performed, and odds ratios were calculated, considering the respondents’ use of modern contraceptive methods. The binary logistic regression included all study variables with a *P* value < 0.20 in the univariate analysis. The significance level was 5%, with 95% confidence intervals (CIs). The Wald and Hosmer-Lemeshow tests were used to assess the significance of independent variables and the goodness-of-fit of the final model, respectively.

### Ethical considerations

This study received ethical approval from the Comité National d'Ethique pour la Recherche en Santé of the Republic of Guinea (No: 045/CNERS/19).

## Results

### Sociodemographic characteristics of care providers and prevalence of FP method use

A total of 1,734 women (aged 20–49) were included in the study. The median age was 29 years, with an interquartile range of (5 24–31). Over half (55.3%) of the women were aged between 25 and 34, and 1,489 (85.9%) worked in public facilities (National/Municipal Hospital and primary health centers). Two-thirds (69.1%) of these women were married. In terms of profession, 838 (48.3%) and 460 (26.5%) of the women were midwives and technical health workers (TWAs), respectively. Moreover, 95.5% of the women had attained a higher level of education, while the remainder had secondary education. Over half (42.5%) of the women had more than 6 years of work experience. Of these providers, nearly 82% had received training in sexual and reproductive health in the twelve months preceding the survey (Table 1).

**Table 1:** Sociodemographic characteristics of female health care providers in the eight (8) administrative regions of Guinea from November 1, 2020, to January 31, 2021 (*n* = 1,734).

Characteristic	Overall	Usage of PFM		
	*N* = 1,734^1^	None	Yes	*p*-value^2^
		*N* = 631^1^	*N* = 1,103^1^	
Natural region				<0.001
Conakry	336.0 (19.4)	176.0 (52.4)	160.0 (47.6)	
Lower Guinea	288.0 (16.6)	41.0 (14.2)	247.0 (85.8)	
Middle Guinea	271.0 (15.6)	114.0 (42.1)	157.0 (57.9)	
Upper Guinea	515.0 (29.7)	117.0 (22.7)	398.0 (77.3)	
Forest Guinea	324.0 (18.7)	183.0 (56.5)	141.0 (43.5)	
Type of health facility				>0.9
National/Municipal Hospitals	465.0 (26.8)	169.0 (36.3)	296.0 (63.7)	
Primary Health Centers	1,024.0 (59.1)	370.0 (36.1)	654.0 (63.9)	
Private Hospitals	245.0 (14.1)	92.0 (37.6)	153.0 (62.4)	
Age	29 (25, 34)	28 (25, 34)	30 (26, 34)	0.052
Age group				<0.001
45 years and more	69.0 (4.0)	39.0 (56.5)	30.0 (43.5)	
20–24 years	303.0 (17.5)	134.0 (44.2)	169.0 (55.8)	
25–34 years	1,010.0 (58.2)	341.0 (33.8)	669.0 (66.2)	
35–44 years	352.0 (20.3)	117.0 (33.2)	235.0 (66.8)	
Statut matrimonial				0.009
No married	535.0 (30.9)	219.0 (40.9)	316.0 (59.1)	
Married	1,199.0 (69.1)	412.0 (34.4)	787.0 (65.6)	
Education level				<0.001
Higher education	1,656.0 (95.5)	625.0 (37.7)	1,031.0 (62.3)	
Secondary education	78.0 (4.5)	6.0 (7.7)	72.0 (92.3)	
Profession				0.2
Physician	66.0 (3.8)	22.0 (33.3)	44.0 (66.7)	
Biologist Or pharmacist	43.0 (2.5)	21.0 (48.8)	22.0 (51.2)	
Mid-wife	838.0 (48.3)	287.0 (34.2)	551.0 (65.8)	
Nurse	327.0 (18.9)	129.0 (39.4)	198.0 (60.6)	
Technical health worker	460.0 (26.5)	172.0 (37.4)	288.0 (62.6)	
Religion				0.015
Christian	505.0 (29.1)	206.0 (40.8)	299.0 (59.2)	
Muslim	1,229.0 (70.9)	425.0 (34.6)	804.0 (65.4)	
Ethnic group				<0.001
Forestier	568.0 (32.8)	223.0 (39.3)	345.0 (60.7)	
Malinké	520.0 (30.0)	162.0 (31.2)	358.0 (68.8)	
Peulh	504.0 (29.1)	207.0 (41.1)	297.0 (58.9)	
Soussou	142.0 (8.2)	39.0 (27.5)	103.0 (72.5)	
Year of experience	5.0 (3.0, 8.0)	4.0 (2.0, 9.0)	5.0 (3.0, 8.0)	0.2
Year of experience				0.13
0–5 years	997.0 (57.5)	378.0 (37.9)	619.0 (62.1)	
6 years and more	737.0 (42.5)	253.0 (34.3)	484.0 (65.7)	
Family planning service				<0.001
None	397.0 (22.9)	197.0 (49.6)	200.0 (50.4)	
Yes	1,337.0 (77.1)	434.0 (32.5)	903.0 (67.5)	
Training over the past 12 months				<0.001
None	1,417.0 (81.7)	546.0 (38.5)	871.0 (61.5)	
Yes	317.0 (18.3)	85.0 (26.8)	232.0 (73.2)	

^1^ *n* (%); median (Q1, Q3).

^2^ Pearson's Chi-squared test; Wilcoxon rank sum test.

### Prevalence of modern contraceptive use

**Figure 2:**
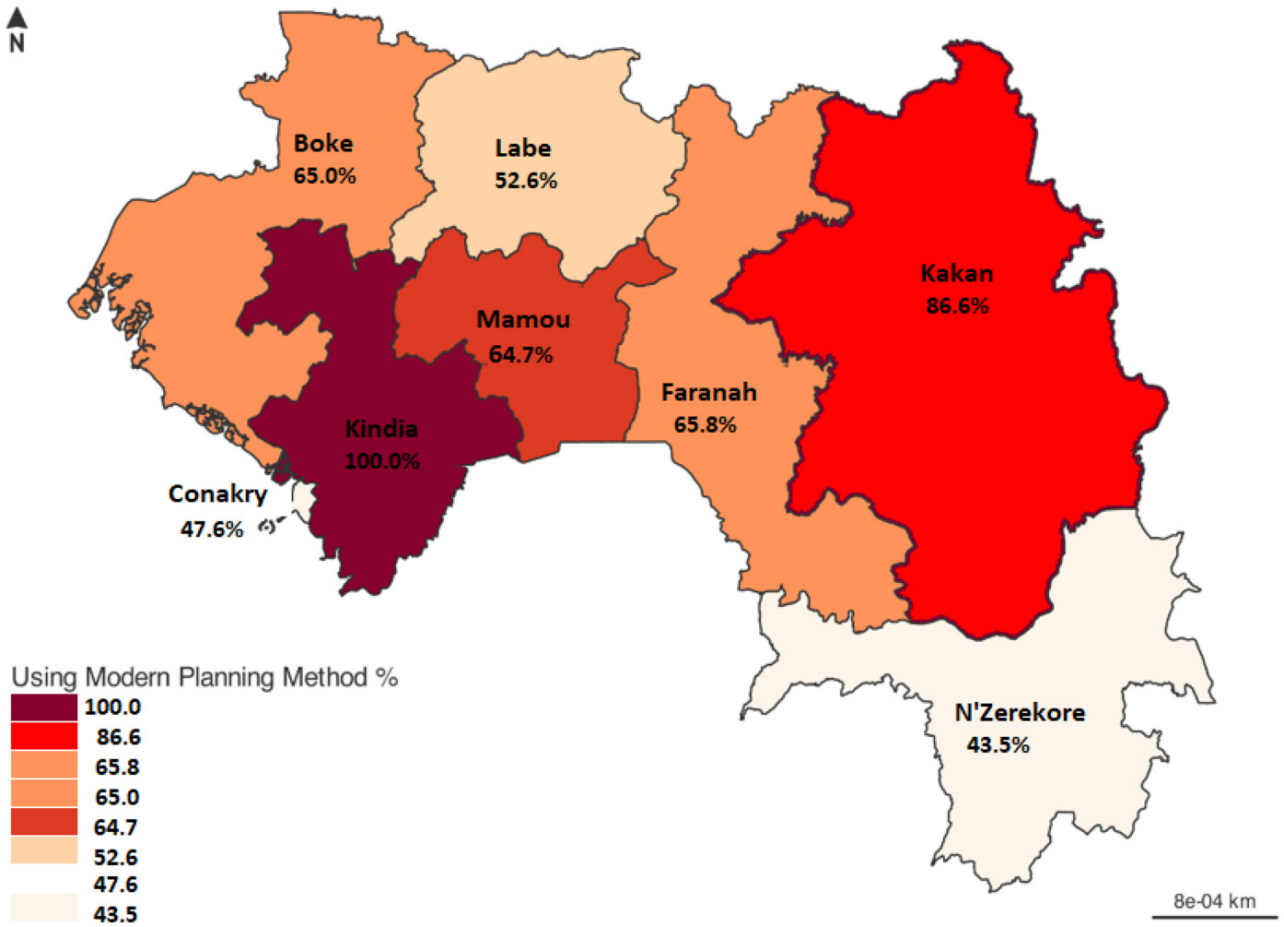
Percentage use of modern contraceptive methods among female health providers from November 1, 2020, to January 31, 2021, in Guinea's eight administrative regions (*n* = 1,734).

In this study, the overall prevalence of modern contraceptive use among female healthcare providers was 61.9% (95%IC: 59.6–64.1). The highest proportions were recorded among married women (64%), those with a higher level of education, those offering FP services (66%) and those who had received SRH training (71.3%) in the last 12 months following data collection. It varies from region to region (Figure 1). The highest prevalence of use of modern contraceptive methods was observed in Kindia (100% of providers), Kankan (86%), Mamou (65%), Faranah (64.9%) and Boké (64.5%) (Figure 2).

The main modern contraceptive methods used by female providers were implants/norplants/jadelle (42%), pills (20%), depoprovera/injectable (16%) and IUD/Sterilet (14%). Male/female condoms (7.9%).

### Factors associated with the use of modern contraceptive methods by female health care providers

In the bivariate analysis, we observed that age, marital status, level of education, religion, working in a planning service, and having received training in the last 12 months prior to data collection were factors significantly associated with the use of modern contraceptive methods among female providers (*p*-value < 0.05).

However, after adjusting for confounding factors, only age, education level, being involved in the provision of modern contraceptive services, having had training in the last 12 months, and religion were statistically significantly higher—the use of modern methods of contraception among female recipients. Indeed, normally for female beneficiaries aged 45 years or less, those aged 25–34 years (aOR: 3.17: 95% CI: 1.85–5.49) and 35–44 years (aOR: 2.81 95% CI: 1.63–4.89) were more likely to use a modern method of contraception. Female providers with a secondary education level (aOR: 7.92; CI: 3.68–20.7) were eight times more likely to use a modern method of contraception than those with a higher level. Similarly, female providers directly involved in providing contraceptive services (aOR: 1.82; CI: 1.41–2.35) were nearly twice as likely to use a modern contraceptive method as their counterparts counterparts (Table 2).

**Table 2:** Factors associated with the use of modern contraceptives by female health care providers in Guinea.

Characteristic	Univariate model			Multivarate model		
	aOR^1^	95% CI^1^	*p*-value	aOR^1^	95% CI^1^	*p*-value
Age group						
45 years and more	—	—		—	—	
20–24 years	1.64	0.97, 2.79	0.066	2.01	1.11, 3.69	0.023
25–34 years	2.55	1.56, 4.21	<0.001	3.17	1.85, 5.49	<0.001
35–44 years	2.61	1.55, 4.44	<0.001	2.81	1.63, 4.89	<0.001
Type of health facility						
National/Municipal Hospitals	—	—				
Primary Health Centers	1.01	0.80, 1.27	>0.9			
Private Hospitals	0.95	0.69, 1.31	0.8			
Marital status						
No married	—	—		—	—	
Married	1.32	1.07, 1.63	0.009	1.19	0.95, 1.50	0.13
Education level						
Higher education	—	—		—	—	
Secondary education	7.27	3.42, 18.8	<0.001	7.92	3.68, 20.7	<0.001
Profession						
Physician	—	—		—	—	
Biologist Or Pharmacist	0.52	0.24, 1.15	0.11	0.76	0.34, 1.70	0.5
Mid-wife	0.96	0.56, 1.62	0.9	0.98	0.56, 1.67	>0.9
Nurse	0.77	0.43, 1.33	0.4	0.94	0.52, 1.66	0.8
Technical health worker	0.84	0.48, 1.43	0.5	0.93	0.52, 1.61	0.8
Religion						
Christian	—	—		—	—	
Muslim	1.30	1.05, 1.61	0.015	1.37	1.09, 1.71	0.006
Year of experience						
0–5 years	—	—		—	—	
6 years and more	1.17	0.96, 1.43	0.13	1.12	0.86, 1.45	0.4
Family planning service						
None	—	—		—	—	
Yes	2.05	1.63, 2.57	<0.001	1.82	1.41, 2.35	<0.001
SRH training in the last 12 months						
None	—	—		—	—	
Yes	1.71	1.31, 2.25	<0.001	1.47	1.10, 1.96	0.009

^1^ aOR, odds ratio; CI, confidence interval.

Female providers who received training in the last 12 months prior to collection were 1.47 times more likely to use modern contraceptive methods. Similarly, Muslim female recipients were 1.37 times more likely to use modern contraceptive methods than their Christian counterparts (Table 2).

## Discussion

This study is one of the first to assess the use of modern contraceptive methods among female health care providers in facilities involved in the provision of family planning services in Guinea. It made it possible to estimate the prevalence and identify the factors associated with this use in women of childbearing age. The prevalence observed in our sample was about 62%, a level significantly higher than that reported in the general female population in Guinea according to the 2018 DHS (11%) (24). This difference can be explained by the very nature of our study population, composed exclusively of health professionals, who are probably more informed about the benefits of modern contraceptive methods. Our results are similar to those reported in Uganda (73.6%) (25) and Southern Africa (69%) (26) but higher than those observed in Ghana (18%) (27).

The most commonly used methods in our study were implants, pills, injectable Depo-Provera, and IUD, which is partially consistent with trends observed in other African contexts, although preferences vary by country (17, 24). A disparity in prevalence was observed between administrative regions, with particularly high use in Kindia and Kankan. These results could be attributed to targeted interventions by technical and financial partners in these regions, including capacity-building sessions and the provision of free family planning services (29).

In terms of associated factors, our analysis found a significant association between educational attainment, participation in the provision of modern contraceptive services, having received training in the past 12 months, and religion and use of modern contraceptive methods. Although training in sexual and reproductive health (SRH) services in the past year was significantly associated with contraceptive use in univariate analysis, this association did not persist in the multivariate model. This result could be explained by the confounding influence of other variables, such as job role or previous exposure to family planning programs. Similar observations have been reported in previous studies, where the initial effect of training decreased after adjusting for sociodemographic and institutional factors that had a stronger predictive value for contraceptive use (30). This highlights the fact that while training remains important, it cannot independently lead to behaviour change unless it is complemented by supportive environments and continuous professional development.

Women under the age of 45 were more likely to use a modern contraceptive method. This can be explained by the socio-cultural context of Guinea, where a majority of women marry young (between 15 and 23 years old), which leads them, after reaching the desired number of children, to use contraception more frequently to space or limit births. This finding is consistent with other studies conducted in Africa and the Maghreb (31, 5).

Regarding educational attainment, a counterintuitive result was observed: women with a secondary education were more likely to use a modern contraceptive method than those with a higher level of education. In our population of health care providers, this could be because women at the secondary level are often midwives or health technicians directly involved in the delivery of family planning services. Their hands-on training and daily exposure to contraceptive methods likely increases their familiarity with and adherence to these methods. It is also possible that awareness campaigns will target more mid-level providers, strengthening their personal commitment to contraceptive use.

### Limitations and strengths of the study

Our study presents a limitation as well as strengths: From a limiting point of view, it should be mentioned that sexuality is a taboo subject for the population. Talking about family planning makes some people hesitant. Therefore, it is not easy to ensure that women provide honest answers to all questions, particularly those relating to the use of contraceptive methods. Furthermore, our study has strengths. It was conducted on a representative sample of all female providers in the country.

## Conclusion

This study shows that more than two-thirds of female healthcare providers use modern contraceptive methods in Guinea, which is generally satisfactory. Factors associated with the use of modern contraceptive methods were young age, secondary education level, religion, women's involvement in family planning services and recent SRH training.

The implications of this study would be to define strategies and interventions focused on the main factors identified. It would be helpful for decision-makers to direct future interventions on the continuing training of providers and emphasize education and awareness campaigns for all stakeholders. The authorities should periodically initiate capacity-building sessions on family planning. They should also think about the One Health aspect by including all staff from all public and private structures as well as NGOs in the implementation of continuing training on SRH and FP.

## Data Availability

The raw data supporting the conclusions of this article will be made available by the authors, without undue reservation.
